# Non-Overlapping Functions for Pyk2 and FAK in Osteoblasts during Fluid Shear Stress-Induced Mechanotransduction

**DOI:** 10.1371/journal.pone.0016026

**Published:** 2011-01-25

**Authors:** Suzanne R. L. Young, Julia M. Hum, Eric Rodenberg, Charles H. Turner, Fredrick M. Pavalko

**Affiliations:** 1 Department of Cellular and Integrative Physiology, Indiana University School of Medicine, Indianapolis, Indiana, United States of America; 2 Department of Biomedical Engineering, Indiana University Purdue University Indianapolis (IUPUI), Indianapolis, Indiana, United States of America; Ecole Polytechnique Federale de Lausanne, Switzerland

## Abstract

Mechanotransduction, the process by which cells convert external mechanical stimuli such as fluid shear stress (FSS) into biochemical changes, plays a critical role in maintenance of the skeleton. We have proposed that mechanical stimulation by FSS across the surfaces of bone cells results in formation of unique signaling complexes called mechanosomes that are launched from sites of adhesion with the extracellular matrix and with other bone cells [1]. Deformation of adhesion complexes at the cell membrane ultimately results in alteration of target gene expression. Recently, we reported that focal adhesion kinase (FAK) functions as a part of a mechanosome complex that is required for FSS-induced mechanotransduction in bone cells. This study extends this work to examine the role of a second member of the FAK family of non-receptor protein tyrosine kinases, proline-rich tyrosine kinase 2 (Pyk2), and determine its role during osteoblast mechanotransduction. We use osteoblasts harvested from mice as our model system in this study and compared the contributions of Pyk2 and FAK during FSS induced mechanotransduction in osteoblasts. We exposed Pyk2^+/+^ and Pyk2^−/−^ primary calvarial osteoblasts to short period of oscillatory fluid flow and analyzed downstream activation of ERK1/2, and expression of c-fos, cyclooxygenase-2 and osteopontin. Unlike FAK, Pyk2 was not required for fluid flow-induced mechanotransduction as there was no significant difference in the response of Pyk2^+/+^ and Pyk2^−/−^ osteoblasts to short periods of fluid flow (FF). In contrast, and as predicted, FAK^−/−^ osteoblasts were unable to respond to FF. These data indicate that FAK and Pyk2 have distinct, non-redundant functions in launching mechanical signals during osteoblast mechanotransduction. Additionally, we compared two methods of generating FF in both cell types, oscillatory pump method and another orbital platform method. We determined that both methods of generating FF induced similar responses in both primary calvarial osteoblasts and immortalized calvarial osteoblasts.

## Introduction

It is well established that mechanical stimulation of bone plays a critical role in maintaining the balance between bone resorption and bone formation. Fluid shear stress (FSS) is generated as a result of interstitial fluid that moves within the bone upon exposure to mechanical stimulation [2]. Osteoblasts respond to this fluid shear stress by controlling expression of proteins involved in bone formation and bone resorption such as cyclooxygenase-2 (COX-2) and prostaglandin E2 (reviewed in [3], [4], [5], [6]) in a process defined as mechanotransduction [7]. Our lab has proposed that changes in gene expression result from unique signaling complexes called mechanosomes that originate at sites of adhesion with the extracellular matrix and with other bone cells [1]. Focal adhesions, which are composed of integrins, vinculin, α-actinin, actin filaments and several other focal adhesion associated proteins, are proposed as likely mechanosensors in bone cells and are ideal launching sites for mechanosomes [8], [9], [10], [11].

Focal adhesion kinase (FAK) is a non-receptor tyrosine kinase that associates with integrins at focal adhesions [12], and association of FAK with integrins at the focal adhesion results in an autophosphorylation event at tyrosine 397 which provides a binding site for Src and other signaling molecules [13], [14]. In addition, the C-terminal domain of FAK can associate with talin and paxillin which connects the focal adhesion with the actin cytoskeleton [15], [16], and the ability of FAK to associated with several downstream effectors makes it a key component of the focal adhesion. Our previous studies reported FAK to function as a part of a mechanosome complex that is required for FSS-induced mechanotransduction in osteoblasts (reviewed in [17]). We demonstrated that FAK^−/−^ osteoblasts fail to appropriately increase the protein levels of COX-2, c-Fos and osteopontin (OPN) in response to oscillatory fluid flow (OFF) [18]. Furthermore, FAK^−/−^ osteoblasts exhibited impaired OFF-induced IκB-β and IκB-α degradation and NF-κB nuclear translocation [19].

Proline- rich tyrosine kinase 2 (Pyk2) is another member of the FAK family of non-receptor tyrosine kinases that can also localize to focal adhesions [20]. FAK and Pyk2 exhibit ∼48% amino acid sequence identity and share a similar domain structure. Both contain a unique N terminus, a protein tyrosine kinase domain and two proline-rich regions at the C terminus [21]. Unlike FAK which is ubiquitously expressed, Pyk2 expression is restricted with the highest levels of expression in the brain and hematopoietic cells [21], [22]. Pyk2 is also highly expressed in osteoclasts in which it is primarily found in podosomes, actin-rich structures that mediate cell attachment and migration [23], [24], [25]. Like FAK, Pyk2 has also been implicated in the regulation of bone health and mechanotransduction. Decreased expression of Pyk2 in murine osteoclast-like multinucleated cells exhibited impaired spreading and inhibited osteoclast bone resorption [26]. Pyk2 can be also be found at focal contacts in ROS 17/2.8 osteoblast-like cells, and Pyk2 moves away from focal contacts in response to cyclic strain, which makes Pyk2 a likely candidate for functioning as part of a mechanosome [27]. Additionally, mechanical strain of ROS 17/2.8 osteoblast-like cells resulted in the autophosphorylation of Pyk2 and FAK [28].

Furthermore, *Pyk2^−/−^* mice exhibit osteopetrosis, a disease in which the bones become very dense and brittle [29], [30]. Gil-Henn *et al*. describe the osteopetrotic phenotype of the *Pyk2^−/−^* mice as a result of defective osteoclast podosome organization and loss of the podosome belt, which leads to reduced bone resorption [29]. Gil-Henn *et al*. further demonstrated that Pyk2 regulates Rho activity in osteoclasts, and loss of this regulation caused defective microtubule- dependent podosome organization. In contrast, Buckbinder *et al.* also described their *Pyk2^−/−^* mice as osteopetrotic, but determined this phenotype was a result of increased bone formation. Buckbinder *et al.* demonstrated normal osteoclast activity in bone marrow derived osteoclasts from *Pyk2^−/−^* mice as determined by TRAP assay [30]. This study also demonstrated an increase in alkaline phosphatase activity and increased bone mineralization in bone marrow cultures from *Pyk2^−/−^* mice and in human mesenchymal stem cells treated either with short hairpin RNA directed against Pyk2 or treated with a catalytically inactive Pyk2 mutant [30]. These studies together indicate the need for further investigation into the function of Pyk2 during bone mechanotransduction.

The goal of our study was to better understand the function of Pyk2 in osteoblasts, specifically analyzing the role Pyk2 plays during osteoblast mechanotransduction. In this article, we analyzed the function of Pyk2 during the immediate early response to FF-induced mechanotransduction in Pyk2^+/+^ and Pyk2^−/−^ primary calvarial osteoblasts to determine if Pyk2, like FAK, functions as part of a mechanosome complex. We employed two methods of generating fluid flow: 1) parallel plate flow chambers and an oscillatory pump, 2) orbital rotating platform, and we then analyzed phosphorylation of ERK1/2, c-Fos protein levels, COX-2 protein levels and OPN expression. We then directly compared these results to the immediate early response of FAK^+/+^ and FAK^−/−^ calvarial osteoblast clones exposed to both methods of FF. We determined that Pyk2, unlike FAK, is not necessary for the immediate early response to FF-induced mechanotransduction in osteoblasts. We also determined that there is not a significant difference between the immediate early response of osteoblasts exposed to FF generated by the orbital rotating platform and osteoblasts exposed to FF generated by parallel plate flow chamber/oscillatory pump system, as analyzed in both Pyk2^+/+^ and Pyk2^−/−^ primary osteoblasts and the FAK^+/+^ and FAK^−/−^ osteoblast clones.

## Materials and Methods

### Ethics Statement

All experiments were performed in accordance with Indiana University's Institutional Animal Care and Use Committee (IACUC). Animals and records of their use were maintained in compliance with the Indiana University HHS Animal Welfare Assurance approval ID #A4091-01. Animals were housed in a light and temperature controlled environment and given food and water ad libitum. Euthanasia was performed using carbon dioxide as an inhalant, and death assured by cardiac puncture in compliance with the guidelines of our Indiana University's Institutional Animal Care and Use Committee. This method is consistent with the recommendations of the Panel on Euthanasia of the American Veterinary Medical Association.

### Cell culture

Osteoblast-like cells were isolated from Pyk2^+/+^ and Pyk2^−/−^ mouse calvaria as described previously [31] and are referred to as osteoblasts throughout the text. Briefly, calvaria from 10-13 neonatal mice (birth to day 3) were isolated aseptically and minced and digested consecutively for 5, 20 and 45 minutes at 37°C in 0.2% collagenase P-0.25% trypsin. All experiments were performed in accordance with Indiana University's Institutional Animal Care and Use Committee (IACUC). Animals and records of their use were maintained in compliance with the Indiana University HHS Animal Welfare Assurance approval ID #A4091-01. Cells released in the second and third digests were pooled and cultured in MEM supplemented with 10% FCS and antibiotics. Experiments were performed with passages 1–3.

Immortalized FAK^−/−^ and FAK^+/+^ osteoblast clones were established as described in Kim et al. 2007. Briefly, primary cells from mouse calvaria were harvested from *fak ^floxed/floxed^*; *p53^−/−^* conditional knockout mouse and infected using CMV-Cre adenovirus to knockout the *fak* gene (Cre^+^; FAK^−/−^; p53^−/−^) [32]. Single cell cloning was performed using a limiting dilution, and individual clones were established as cell lines. Control calvarial osteoblasts harvested from *fak ^floxedfloxed^* mouse that were not exposed to Cre recombinase (Cre^−^; FAK^floxed/floxed^; p53^−/−^) were established in a similar fashion [32]. The mouse clones were cultured in MEM supplemented with 10% FCS and antibiotics. Both FAK^+/+^ and FAK^−/−^ clones express the osteoblast marker osteopontin and alkaline phosphatase and contribute to fetal skeletogenesis *in vivo* [32].

### Oscillatory fluid flow conditions

Osteoblasts were plated on glass slides at ∼2.0×10^5^ cells per slide (∼1.1×10^5^ cells/cm^2^). Cells were subjected to oscillatory fluid shear stress (∼10 dynes/cm^2^) in parallel plate flow chambers at 37°C using a previously described fluid flow device [33]. Hard-walled tubing was used to connect the pump to the chamber inlet, and a reservoir was attached to the outlet to allow for movement of the fluid and exchange of 5% CO_2_. This system subjects cells to oscillating fluid flow at a frequency of 0.5 Hz in MEMα supplemented with 0.5% FCS and antibiotics. Static controls were held in cell culture dishes at 37°C with 5% CO_2_ also in MEMα supplemented with 0.5% FCS and antibiotics.

### Orbital rotating platform conditions

Osteoblasts were plated into individual wells of 6 well plates at ∼1.2×10^5^ cells per well (∼1.1×10^5^ cells/cm^2^) in MEMα supplemented with 0.5% FCS and antibiotics. Cells were subjected to dynamic fluid flow generated by 1 ml of media on an orbital platform shaker rotating at a speed of ∼200 rpm (2 Hz) inside a tissue culture incubator at 37°C with 5% CO_2_. Static controls plates were held at 37°C with 5% CO_2_ also in MEMα supplemented with 0.5% FCS and antibiotics.

### Immunoblot analysis

Cells subjected to static and fluid flow conditions were harvested in SDS sample buffer, and protein concentrations were determined using the amido black method [34]. Equal amounts of protein (20 µg) were loaded onto SDS-PAGE gels for separation and transferred to PVDF (nitrocellulose was used when probing for phospho-Pyk2). The following primary and secondary antibodies were used: ERK1/2 (Santa Cruz), phospho-ERK1/2 (Santa Cruz), COX-2 (Santa Cruz), c-Fos (Santa Cruz), FAK (Upstate), Pyk2 (BD), vinculin (Sigma), HRP conjugated donkey anti-rabbit and HRP conjugated donkey anti-goat (Jackson Immunoresearch, West Grove, PA), HRP conjugated donkey anti-goat (Santa Cruz). Densitometry was quantitated using Image J software (NIH).

### RNA Extraction, cDNA synthesis and quantitative real-time PCR (qRT-PCR) analysis

To harvest RNA from cultured osteoblasts, cells were harvested in Trizol® (Invitrogen, Carlsbad, CA), and RNA was extracted with chloroform and precipitated with isopropanol. First strand cDNA synthesis was performed using M-MLV reverse transcriptase (Promega). Real-time PCR primers were designed for osteopontin (OPN accession no. BC057858) and GAPDH (accession no. BC096042). OPN Forward: 5′ AATGCTGTGTCCTATGAAAA; OPN Reverse: 5′TCGACTGTAGGGACGATTGGA; GAPDH Forward: 5′ GGCCGAGAATGGGAAGCAGG ; GAPDH Reverse: 5′ GGGGTGGGTGGTCAAGGGTT. We used SYBR Green Fast Start Master Mix (Roche) amplification in an ABI 7500 real-time PCR system (Applied Biosystems). The amplification conditions were as follows: 15 min at 95°C; 15 sec at 95°C; 40 sec at 60°C; 40 cycles of 15 sec at 95°C; 40 sec at 60°C; dissociation. The ΔΔCT method was used to evaluate gene expression between samples (using GAPDH as a loading control).

### Statistical analysis

Statistical significance was assessed by either two-tailed *t-test* or a two way analysis of variance (ANOVA) with a *p*-value of p<0.05 interpreted as statistically significant.

## Results

### Pyk2 deficient osteoblasts exhibit increased c-Fos protein levels in response to Fluid Flow

Two approaches were employed to evaluate the roles of FAK and Pyk2 during FF-induced mechanotransduction in osteoblasts. First, we exposed osteoblasts plated on glass slides at a density of ∼1.1×10^5^ cells/cm^2^ to oscillatory fluid flow (OFF) using the parallel plate flow chambers as described in the Methods section. As a second, alternative method we plated cells at the same density (∼1.1×10^5^ cell/cm^2^) in each well of a 6 well plate. We then placed the 6 well plates on an orbital rotating platform at a speed of ∼200 rpm (see also Methods) to generate dynamic fluid flow. A direct comparison of these two methods was performed using a two-tailed *t-test* or an analysis of variance.

**Figure 1:**
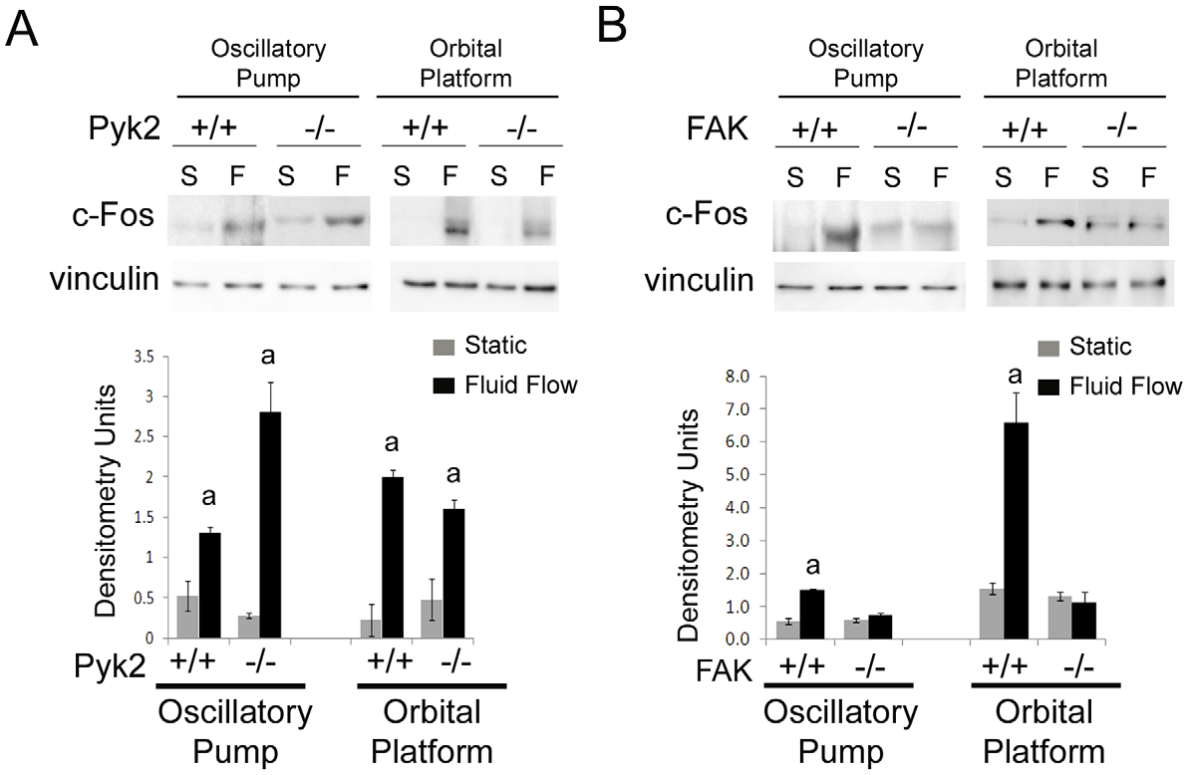
Analysis of c-Fos protein levels in response to Fluid Flow (FF). (A) Western blot analysis of c-Fos protein in Pyk2^+/+^ and Pyk2^−/−^ osteoblasts exposed to 30 minutes of FF (F) or maintained in static culture conditions (S). (B) Western blot analysis of c-Fos protein in FAK^+/+^ and FAK^−/−^ osteoblasts exposed to 30 minutes of FF (F) or maintained in static culture conditions (S). Vinculin was analyzed as a loading control. Graph represents quantification of c-Fos protein levels expressed as raw densitometry units. Experiments were performed in triplicate, and n≥3 for each experimental group in each trial. Error bars represent standard error. ^a^Statistically significant difference between static and FF (p<0.05).

In osteoblasts, c-Fos is up-regulated upon stimulation of short periods of fluid flow (FF) and may play a role in bone remodeling by transcriptional activation of matrix proteins [35], [36], [37], [38], [39], [40], [41]. Osteoblasts harvested from calvarial of *Pyk2^+/+^* and *Pyk2^−/−^* mice were plated as above on glass slides or in 6 well plates and exposed to both methods of FF for 30 minutes or maintained in static culture conditions. Western blot analysis of c-Fos protein was performed. Both Pyk2^+/+^ and Pyk2^−/−^ osteoblasts exhibited a significant 2–8 fold increase in c-Fos protein upon exposure to FF generated by the oscillatory pump system as compared to static controls (Fig. 1A). Similarly, both of Pyk2^+/+^ and Pyk2^−/−^ exhibited a significant 2-10 fold increase when exposed to FF generated by the orbital platform. There was not a significant difference between Pyk2^+/+^ and Pyk2^−/−^ osteoblasts response to FF generated by either method. Furthermore, there was not a significant difference between the two methods in the fold increase in c-Fos protein levels.

FAK^+/+^ and FAK^−/−^ osteoblast clones were also exposed to both methods of FF for 30 minutes or maintained in static culture conditions. Western blot analysis of c-Fos protein was performed, and FAK^+/+^ osteoblasts exhibited a significant 1.6–2.7 fold increase in c-Fos protein upon exposure to FF generated by the oscillatory pump system as compared to static controls (Fig. 1B). Additionally, FAK^+/+^ osteoblasts exhibited a significant 1.7-4.3 fold increase in response to FF generated by the orbital platform. FAK^−/−^ osteoblasts did not exhibit a significant increase in c-Fos protein in response to either method of FF. As above, there was not a significant difference between the fold difference in c-Fos proteins levels in FAK^+/+^ osteoblasts using either method of FF (Fig. 1B).

### Appropriate FF induced ERK phosphorylation in Pyk2 deficient osteoblasts

**Figure 2:**
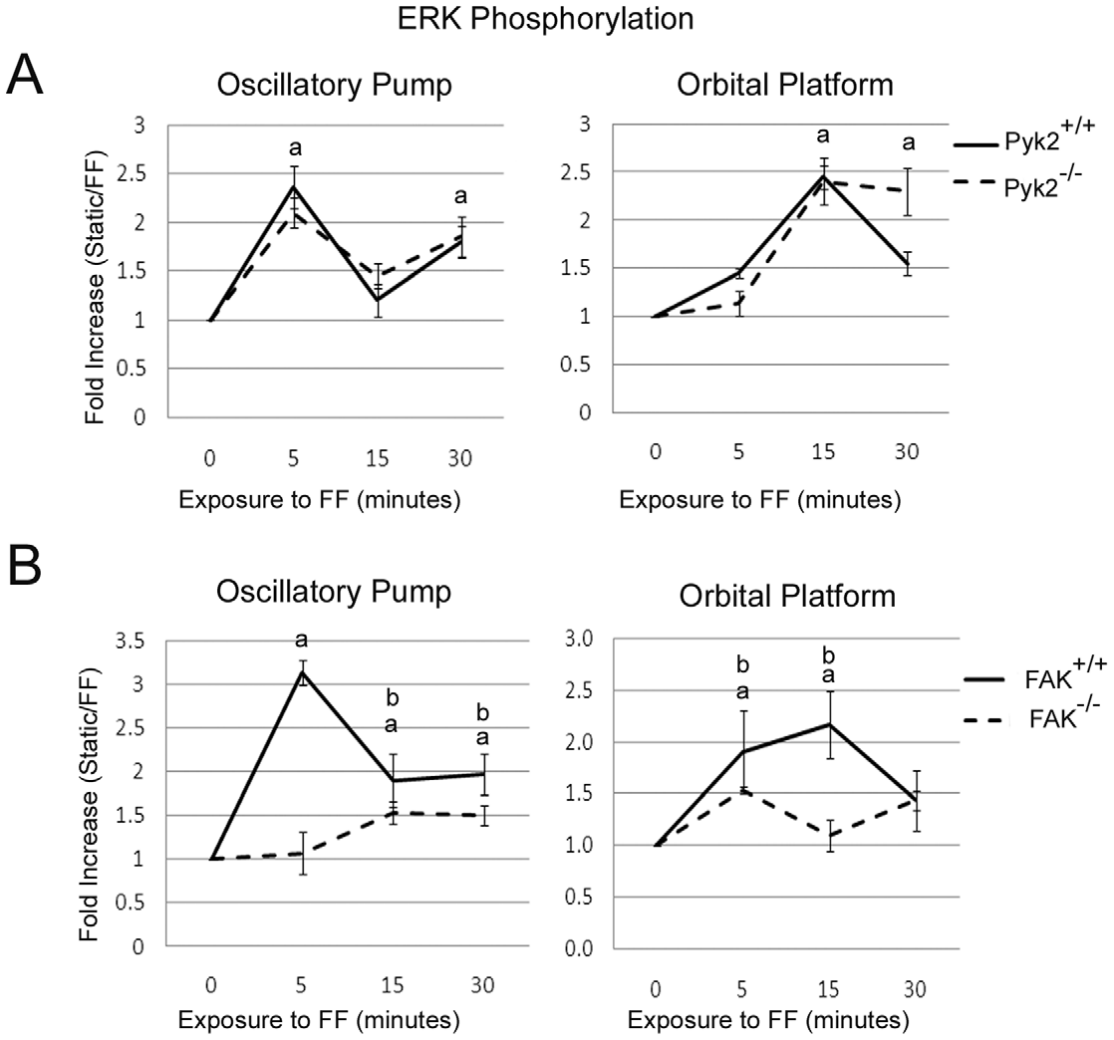
Appropriate FF induced ERK1/2 phosphorylation in Pyk2^−/−^ osteoblasts. (A) Graphical representation of western blot analysis of ERK1/2 phosphorylation in Pyk2^+/+^ and Pyk2^−/−^ osteoblasts exposed to 5, 15 and 30 minutes of FF (F) or maintained in static culture conditions (S). (B) Graphical representation of western blot analysis of ERK1/2 phosphorylation in FAK^+/+^ and FAK^−/−^ osteoblasts exposed to 5, 15 and 30 minutes of FF (F) or maintained in static culture conditions (S). Experiments were performed in triplicate, and n≥3 for each experimental group in each trial. Error bars represent standard error. ^a^Statistically significant difference between static and FF (p<0.05). ^b^Statistically significant difference between FAK^+/+^ and FAK^−/−^ (p<0.05).

To further determine the role of Pyk2 during osteoblast mechanotransduction after short periods of FF, we analyzed ERK signaling. ERK becomes phosphorylated in osteoblasts within 5 minutes of FF and begins to decline after 30 minutes of FF [42], [43], [44], [45], [46], [47], [48], [49]. We examined phosphorylation of ERK in response to 5, 15 and 30 minutes of FF by western blot analysis. Both Pyk2^+/+^ and Pyk2^−/−^ osteoblasts exhibited a significant 2.0–2.8 fold increase in phosphorylated ERK after exposure to 5 minutes of FF generated by the oscillatory pump as compared to static controls (Fig. 2A). Additionally, a significant 1.6–2.3 fold increase was observed after 30 minutes of FF in both Pyk2^+/+^ and Pyk2^−/−^ osteoblasts. Using the orbital platform method, we observed a significant 2.1–2.7 fold and 1.6–2.3 fold increase in phosphorylated ERK after exposure to 15 and 30 minutes of FF, respectively. While Pyk2^+/+^ and Pyk2^−/−^ osteoblasts did not exhibit a difference in their response to FF using either method, we did observe a difference in the timing of ERK phosphorylation between the two methods.

We observed a significant 2.9–3.4 fold, 1.5–2.5 fold and 1.6–2.4 fold increase in ERK phosphorylation in FAK^+/+^ osteoblasts in response to 5, 15 and 30 minutes of FF generated by the oscillatory pump (Fig. 2B). FAK^−/−^ osteoblasts exhibited a small but significant ∼1.5 fold increase in ERK phosphorylation in response to 15 minutes of FF using the oscillatory pump. However, this response was significantly lower than that of the FAK^+/+^ osteoblasts. Similarly to the results above, using the orbital platform method of generating FF resulted in the apex of ERK phosphorylation (1.8–2.8 fold increase) to occur at 15 minutes in FAK^+/+^ osteoblasts. Additionally, both FAK^+/+^ and FAK^−/−^ osteoblasts exhibited a significant increase at 5 minutes, but again, the response was significantly lower in the FAK^−/−^ as compared to the FAK^+/+^ osteoblasts.

### Pyk2 is not important for increased COX-2 protein levels in response to FF

**Figure 3:**
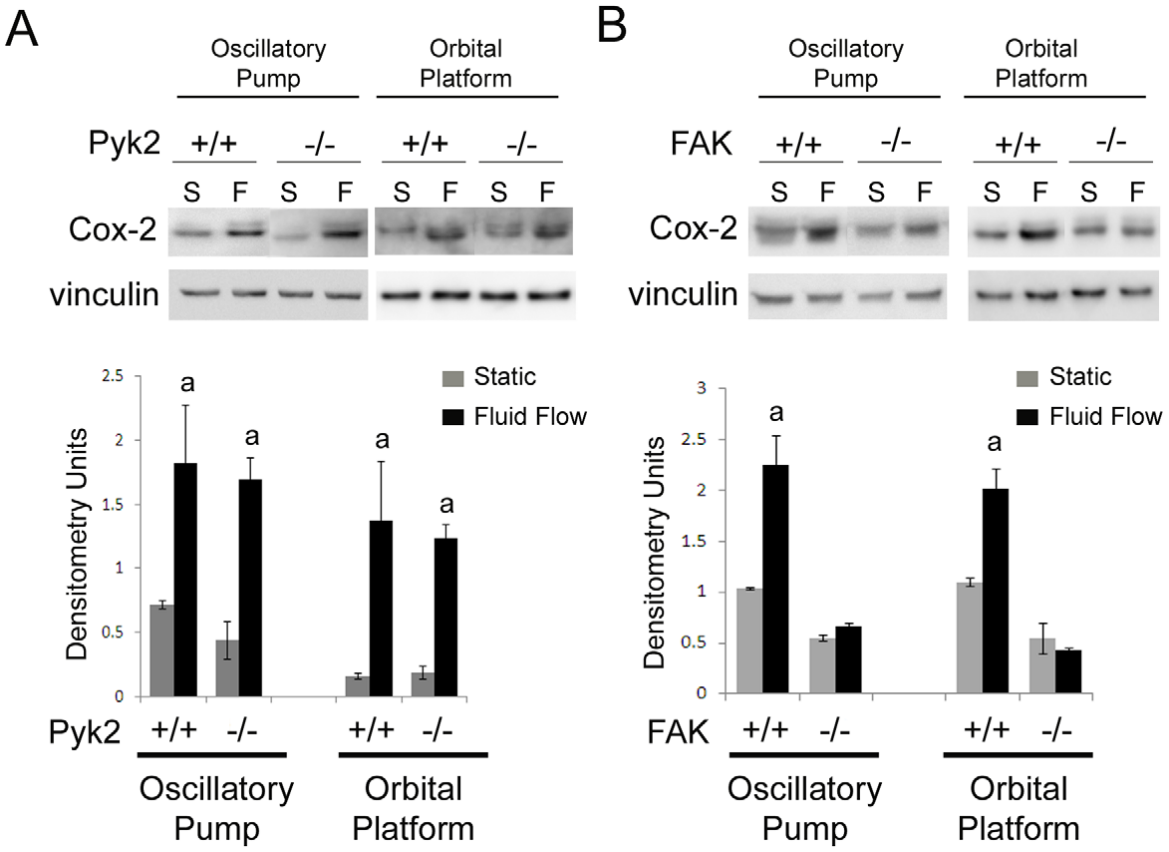
Analysis of COX-2 protein levels in response to FF. (A) Western blot analysis of COX-2 protein levels in Pyk2^+/+^ and Pyk2^−/−^ osteoblasts exposed to either 1 hour of FF followed by a 3 hour post-flow incubation (F) or maintained in static culture conditions (S). (B) Western blot analysis of COX-2 protein levels in FAK^+/+^ and FAK^−/−^ osteoblasts exposed to 2 hours of FF (F) or maintained in static culture conditions (S). Vinculin was analyzed as a loading control. Graphs represent quantification of c-Fos protein levels expressed as raw densitometry units. Experiments were performed in triplicate, and n≥3 for each experimental group in each trial. Error bars represent standard error. ^a^Statistically significant difference between static and FF (p<0.05).

Osteoblasts exhibit increased COX-2 protein levels and increased release of PGE_2_ in response to exposure to longer periods of FF (2–4 hrs), which stimulates bone formation by promoting osteoblast differentiation [39], [50], [51], [52], [53], [54]. We examined the ability of Pyk2 deficient osteoblasts to regulate COX-2 protein levels by exposing osteoblasts to 1 hour of FF followed by a 3 hour post-flow incubation or maintained the cells in static culture conditions. Western blot analysis was performed. Pyk2^+/+^ and Pyk2^−/−^ osteoblasts exhibited a significant 2–4 fold and 3–8 fold increase in COX-2 protein respectively upon exposure to FF generated by the oscillatory pump system as compared to static controls (Fig. 3A). We also determined that both Pyk2^+/+^ and Pyk2^−/−^ osteoblasts exhibited a significant 2–8 fold increase in COX-2 protein in response to FF generated by the orbital platform. There was not a significant difference in the response of COX-2 protein levels between Pyk2^+/+^ and Pyk2^−/−^ osteoblasts using either method. Furthermore, there was not a significant difference between methods in the upregulation of COX-2 protein levels in either Pyk2^+/+^ and Pyk2^−/−^ osteoblasts.

To analyze the up-regulation of COX-2 protein levels in FAK^+/+^ and FAK^−/−^ osteoblast clones, we exposed cells to both methods of FF for 2 hours or maintained cells in static culture conditions. Western blot analysis of COX-2 protein was performed. We determined that FAK^+/+^ osteoblasts exhibited a significant 1.6–2.2 fold increase in COX-2 protein upon exposure to FF generated by the oscillatory pump system as compared to static controls (Fig. 3B). Additionally, FAK^+/+^ osteoblasts exhibited a significant 1.5–2.0 fold increase in response to FF generated by the orbital platform. FAK^−/−^ osteoblasts did not exhibit a significant increase in COX-2 protein in response to either method of FF (Fig. 3B). There was not a significant difference between the fold increase in COX-2 proteins levels in FAK^+/+^ osteoblasts using either method of generating FF.

### FF induced up-regulation of OPN is reduced in FAK deficient osteoblasts

**Figure 4:**
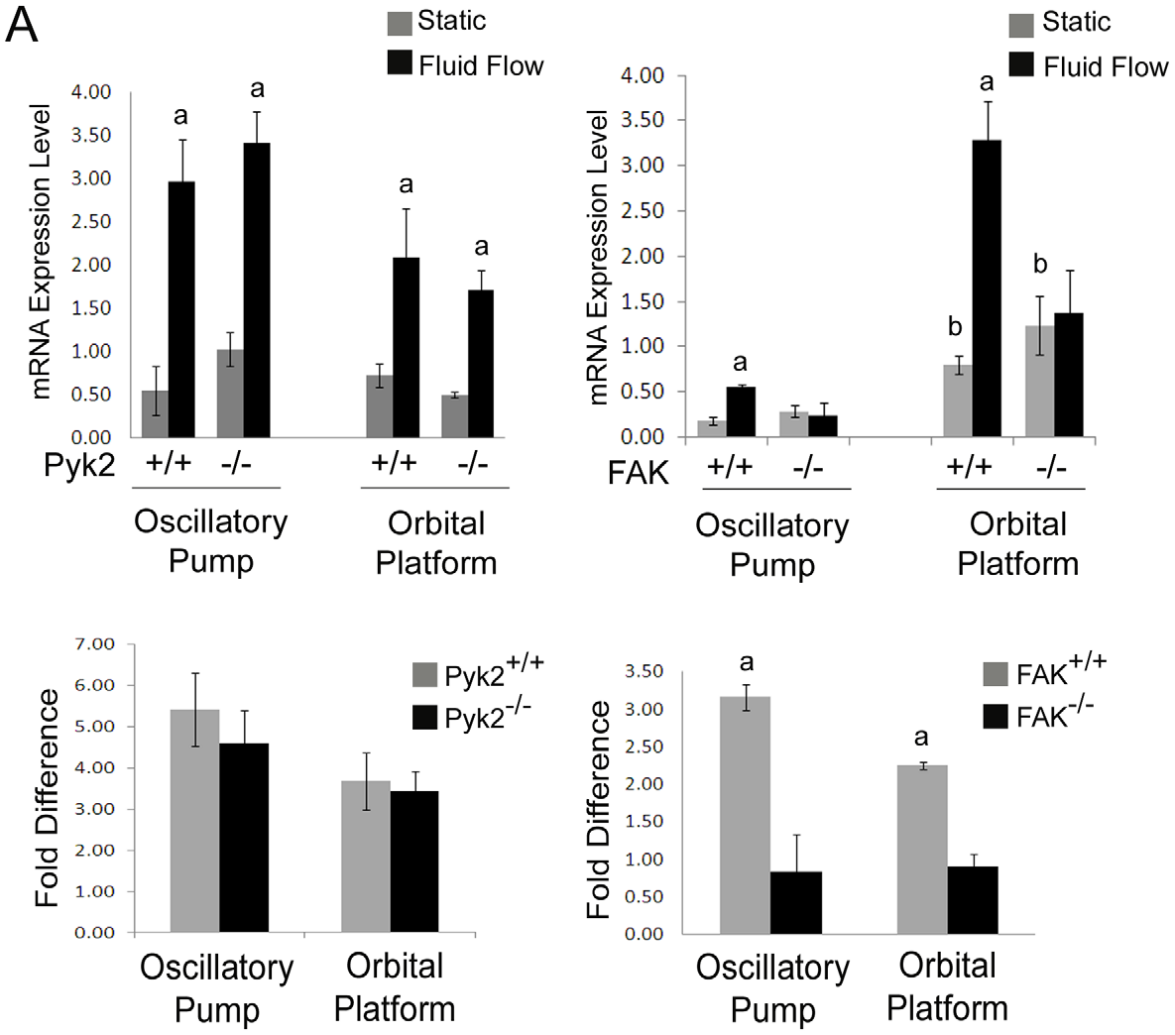
Analysis of OPN expression in osteoblasts exposed to FF. (A) Expression of OPN mRNA as determined by qRT-PCR analysis of Pyk2^+/+^ and Pyk2^−/−^ osteoblasts exposed to either 2 hour of FF followed by a 20 hour post-flow incubation or maintained in static culture conditions. (B) Expression of OPN mRNA as determined by qRT-PCR analysis of FAK^+/+^ and FAK^−/−^ osteoblasts exposed to either 2 hour of FF followed by a 20 hour post-flow incubation or maintained in static culture conditions. (C) Graphical representation of data in Fold Difference. Experiments were performed in triplicate, and n≥3 for each experimental group in each trial. Error bars represent standard error. ^a^Statistically significant difference between static and FF (p<0.05). ^b^Statistically significant difference between static conditions (p<0.05).

OPN is a protein involved in bone remodeling, and its expression is increased in osteoblasts exposed to FF followed by an overnight incubation [38], [48], [55]. We examined the ability of osteoblasts to increase expression of OPN in Pyk2 deficient cells. Osteoblasts were exposed to 2 hours of FF or maintained in static culture conditions then incubated for an additional ∼20 hours upon which time RNA was harvested. First strand cDNA was generated as indicated in the Methods. Analysis of OPN mRNA expression as well as GAPDH mRNA as loading control was performed. Both Pyk2^+/+^ and Pyk2^−/−^ osteoblasts exhibited a significant 4.5–5.5 fold increase in OPN expression in response to FF generated by the oscillatory pump method (Fig. 4A). Both Pyk2^+/+^ and Pyk2^−/−^ osteoblasts exhibited a significant 3.5–4.0 fold increase in OPN expression using the orbital platform method of generating FF. There was not a significant difference between Pyk2^+/+^ and Pyk2^−/−^ osteoblasts in response to FF. Furthermore, there was not a significant difference between the two methods in the fold increase of OPN expression.

As above, we exposed FAK^+/+^ and FAK^−/−^ osteoblasts to 2 hours of FF or maintained cells in static culture conditions both of which were followed by a ∼20 incubation. RNA was harvested and first stand cDNA was generated as indicated in the Methods. Analysis of OPN mRNA expression as well as GAPDH mRNA as loading control was performed. FAK^+/+^ osteoblasts exhibited a significant 3.2–6.4 fold increase in OPN expression upon exposure to FF generated by the oscillatory pump system as compared to static controls (Fig. 4B). FAK^+/+^ osteoblasts also exhibited a significant 2.3–3.2 fold increase in OPN expression in response to FF generated by the orbital platform. FAK^−/−^ osteoblasts did not exhibit a significant increase in OPN expression in response to FF generated by either method. While we did not find a significant difference between the two methods to increase OPN expression, we did find a significant 4–10 fold difference in the basal expression of OPN between osteoblasts plated on glass slides (oscillatory pump method) versus those plated on plastic 6 well plates (orbital platform method) in both FAK^+/+^ and FAK^−/−^ osteoblasts.

## Discussion

The goal of this study was to better understand the role of the FAK and Pyk2 tyrosine kinases during osteoblast mechanotransduction. This work is the first to examine the role of Pyk2 during FF induced mechanotransduction and compare it to the role of FAK. Our results demonstrate that unlike FAK, Pyk2 does not play a critical role during the immediate response to FF-induced mechanotransduction in osteoblasts. We show that Pyk2 deficient osteoblasts, unlike FAK deficient osteoblasts [18], [19], exhibited appropriate increases in c-Fos and COX-2 protein levels, increases in phosphorylation of ERK and appropriate increases in OPN expression in response to FF. Additionally, we have compared two methods of generating FF (oscillatory pump method and the orbital platform method) and determined that these two methods induce a similar response in both primary calvarial osteoblasts and immortalized calvarial osteoblast clones.

Pyk2 is responsive to mechanical stimulation in osteoblasts [27], [28], and it is important for appropriate osteoclast function [26]. Additionally, *Pyk2^−/−^* mice are osteopetrotic [29], [30]. Our lab has demonstrated that FAK, a Pyk2 related protein, is important for FSS induced mechanotransduction in osteoblasts [18], [19]. These data combined lead us to hypothesize that Pyk2 is key component of bone remodeling and maybe required for osteoblast mechanotransduction. However, our study determined that Pyk2 is not critical for the immediate response of osteoblasts to FF, and importantly, does not have an over-lapping function with FAK. Although our data does not support our original hypothesis, it does suggest that Pyk2 may play an alternative role to FAK during FF-induced mechanotransduction.

Our previous work has clearly demonstrated that FAK is key component of FF- induced mechanotransduction in osteoblasts during short periods of mechanical stimulation. This study further demonstrates the importance of FAK during the response of osteoblasts to short periods of FF, and it supports the idea that FAK functions in a mechanosome signaling complex (reviewed in [17]). Additionally, our data indicates that FAK and Pyk2 do not have redundant functions in osteoblasts leading us to hypothesize that Pyk2 may function during long-term mechanical stimulation of osteoblasts. Perhaps Pyk2 and FAK work in concert to regulate both short and long periods of FF in osteoblasts. We suggest that Pyk2 may still be involved in mechanosome signaling complexes because it shuttles away from the membrane and to the nucleus in response to mechanical stimulation [27]. While we currently do not have data to directly support this hypothesis, we are eager to test the role of Pyk2 during long term mechanical stimulation.

While our work indicates that Pyk2 is not involved in the immediate response to FF, we have yet to determine if Pyk2 is involved in mechanically induced osteoblast differentiation, which requires periods of FF over the span of several days. Buckbinder *et al.* demonstrated an increase in alkaline phosphatase activity and increased bone mineralization in bone marrow cultures from *Pyk2^−/−^* mice, which indicates that Pyk2 may be involved in the regulation of differentiating pre-osteoblasts [30]. These data together with the osteopetrotic phenotype of the *Pyk2^−/−^* mice suggest that Pyk2 may be involved in regulating mechanically induced osteoblast differentiation in a mechanosome signaling complex, and we are in the process of testing this hypothesis.

Prior to this study, we have been unable to examine the role of Pyk2 during long periods of FF over the course of several days because of the limiting abilities of the oscillatory pump/parallel plate flow chamber method. Specifically, this method is very difficult to keep sterile for several days. Additionally, we were concerned that the 300 µl volume of media in the chamber was not adequate for the respiration and nutrient exchange needs of the cells over the course of several hours or days. This led us to test the orbital platform method. The orbital platform method puts to use an orbital platform shaker and 6 well plates instead of the parallel plate chambers and glass slides. It is easily kept sterile and requires 1 ml of media, which is adequate for nutrient and gas exchange over the span of several hours and days. Our study compared the oscillatory pump method to the orbital platform method in both the Pyk2 primary calvarial osteoblasts and the FAK calvarial osteoblast clones. We determined that there was not a significant difference between the methods as measured by the response of osteoblasts to up-regulate OPN expression and to increase c-Fos and COX-2 protein levels.

We did observe a difference between the two methods when analyzing the phosphorylation of ERK over time. Using the oscillatory pump methods, we determined that in both primary calvarial osteoblasts and in osteoblast clones the peak of ERK phosphorylation occurred at 5 minutes of FF. Using the orbital pump method we observed this peak in ERK phosphorylation to occur at 15 minutes. Because the oscillatory pump methods requires more manipulation of the glass slide in media while being placed in the parallel plate flow chamber, we suggest that these osteoblasts are being stimulated for approximately 10 minutes prior to turning on the pump. In contrast, the osteoblasts on the 6 well plates are not manipulated prior to starting the orbital platform shaker. Therefore, the extra manipulation of the slides may be a possible reason why we observe a difference in timing of ERK phosphorylation between the two methods. Interestingly, we found that the fold difference at apex of ERK phosphorylation in not significantly different between the two methods. Secondly, the oscillatory pump experiments were conducted at 0.5 Hz while the orbital platform experiments were performed at 2 Hz, which may also contribute to the difference we observe in ERK phosphorylation between the two methods. Finally, the oscillatory pump method generates oscillatory fluid flow while the orbital platform method generates dynamic fluid flow. The differing types of FF could also be a possible explanation for the differences in ERK phosphorylation we observed.

In our study we did observe a difference in OPN expression in immortalized calvarial osteoblasts when placed on glass slides as compared to those plated onto 6 well plates. There was a significant 4–10 fold increase in OPN expression when osteoblasts were plated on 6 well plates (plastic) as compared to cells plated on glass slides. However, we did not observe this increase in OPN expression in the primary calvarial osteoblasts. Therefore, we speculate that the increased OPN expression observed in the immortalized osteoblasts may be due to a clonal effect, though this hypothesis has not yet been tested.

Other labs have also used an orbital platform –like method to mechanically stimulate cells. Kido *et al.* mechanically stimulated osteoblasts using a horizontal shaking apparatus fixed inside a tissue culture incubator to study how mechanical stimulation affects interleukin-11 expression [56]. They estimated that the shear stress force applied to osteoblasts was a little less than ∼2 Pa, which is similar to the amount of shear stress applied to cells using the oscillatory pump method (∼1 Pa). Kalogeropoulos *et al.* also used an orbital shaker to mechanically stimulate osteoblast-like cells (MC3T3-E1) while studying the role of Zic 1 during osteocyte mechanotransduction [57]. Kalogeropoulos *et al.* indicates that the shear stress applied to the cells was ∼7.5 dynes/cm^2^ (∼0.75 Pa). A third study by Zhou *et al.* puts to use a see-saw like shaker to generate fluid shear stress at ∼0.9 dynes/cm^2^ (∼0.09 Pa) [58]. They conclude that this method generates FSS that is similar to a low-magnitude, oscillatory FSS generated by the parallel-plate system. Our study further indicates in a direct comparison, that the orbital platform method can be used to generate a similar level of shear stress that is physiologically appropriate for stimulating osteoblasts.

In summary, we have shown that Pyk2 is not required in osteoblasts for the immediate mechanical response to short periods of FF. Unlike FAK deficient osteoblasts, Pyk2 deficient osteoblasts exhibit appropriate increased c-Fos and COX-2 protein levels, increased OPN expression and increased ERK phosphorylation in response to FF. These data are the first to indicate that FAK and Pyk2 have non-overlapping functions during osteoblast mechanotransduction. Additionally, we have demonstrated that the orbital platform method of generating FF is similar to the oscillatory pump method in both primary and immortalized calvarial osteoblasts as measured by these immediate early responses.
